# Oral health disparities in the elderly: A study on access to dental care and its implications

**DOI:** 10.6026/973206300210832

**Published:** 2025-04-30

**Authors:** Saurabh Sinha, Charu Chitra Govindan, Anjali Sharma, Pramod Kumar Yadav, Amuthavalli E., Anubhuti Jain

**Affiliations:** 1 Department of Dentistry, K M C Medical College, Maharajganj Uttar Pradesh, India; 2 Department of Public Health Dentistry, Rama Dental College, Hospital and Research Centre, Kanpur, Uttar Pradesh, India; 3 Department of Dental Surgery, VMMC and Safdarjung hospital, New Delhi, India; 4 Department of Periodontia & Community Dentistry, Dr Z.A Dental College, AMU, Aligarh, Uttar Pradesh, India; 5 Department of Dental Surgery, K.A.P. Visawanatham Government Medical College, Trichy, Tamil Nadu, India; 6 Department of Public Health Dentistry, Chhattisgarh Dental College and Research Institute, Rajnandgaon, Chhattisgarh, India

**Keywords:** Oral health, elderly, dental care access, disparities, DMFT index, rural-urban comparison

## Abstract

Elderly people encounter important dental health effects on their general well-being even though their access to dental services remains unequal. Seniors living in rural areas showed more tooth decay on dental examination with DMFT scores at 14.2 for of 300 seniors while urban seniors had scores at 9.5. Regular check-ups were accessed by 57% of urban senior citizens whereas only 28% of rural elderly received such visits. The cost of transportation and insufficient funds stood as main obstacles that prevented seniors from attending dental appointments. Enhanced accessibility combined with increased awareness constitutes the base for decreasing healthcare inequalities affecting aging adults' oral health.

## Background:

The dental health of elderly adults maintains fundamental importance to general health and life quality because their susceptibility to dental problems grows due to natural age-related physical modifications and persistent health conditions [1]. A large number of elderly patients still face unresolved dental problems that include dental caries and periodontitis and edentulism because treatment is unavailable to them [2, 3]. The availability of dental care services for elderly patients depends on their socioeconomic conditions along with their location and insurance status and their health-related abilities (body and mind) [4]. Residents who live in rural areas together with underserved populations encounter multiple obstacles including difficult traveling to dental appointments and sparse provider availability and inadequate dental health knowledge [5, 6]. Research indicates that older adults who earn minimum income without dental insurance coverage avoid preventive dental care sessions yet experience increasing negative oral health results [7]. Systemic factors affecting the public health infrastructure along with insufficient integration of dental care services into primary healthcare delivery systems for elderly individuals create additional oral health disparities [8]. The increasing elderly population worldwide requires immediate attention to health inequalities which will help the elderly experience healthy aging alongside decreased oral diseases. The condition of elderly oral health represents both a dental situation and a substantial public health matter since it develops connections with systemic health problems and heart disease and diabetes and breathing infections and inadequate nourishment [9, 10]. Age-related deterioration of oral health increases the importance of both directions in oral and general health because weak oral abilities make nutrition impossible while worsening diseases [11]. Those older adults with dementia or Alzheimer's disease along with cognitive impairment face additional barriers to managing daily oral hygiene which speeds up oral disease progression [12]. Research focuses on three elements which determine how well elderly adults maintain their oral hygiene: health literacy and self-care abilities and family caregiver involvement [13, 14]. Structures of health promotion developed for specific groups and deliverable through educational approaches and community-based programs show promise to enhance oral health outcomes and behavior changes for this population [15]. Public health policies need to focus on multiple oral health factors that create inequalities because these factors drive effective solutions for targeted health interventions. The investigation of dental services access effects on elderly oral health across distinct settings generates necessary evidence for creating equitable and inclusive oral health strategies for senior populations. Therefore, it is of interest to investigate oral health disparities among elderly people in varied regions along with their dental service access impacts on their oral health status.

## Materials and Methods:

A six-month observational cross-sectional research investigated how dental care accessibility affects senior citizens' oral health disparities. Three hundred elderly participants between 65 years and above participated for this study by implementing stratified random sampling across urban and rural locations. The study required participants who surpassed 65 years of age along with the ability for consent and willingness to take part. Subjects with cognitive disabilities or severe systemic conditions making regular oral examination impossible were excluded from the study participation. A structured questionnaire together with clinical oral examination served as the data collection tools. The research instrument utilized for data collection contained questions about participant demographics (age, gender, education level and earnings) and dental care usage patterns in addition to oral hygiene routines, barriers experienced during dental service utilization, dental insurance availability.

The pre-tested and validated questionnaire underwent testing at 20 locations yet excluded from ultimate study analysis. The study assessments took place under natural daylight conditions through the use of disposable probes alongside mirrors. The examination of oral health status depended on assessment through the decayed, missing and filled teeth (DMFT) index. A reliability test for consistency (kappa score = 0.85) was performed before the examiners started measuring to maintain reliable results within the study. The study received ethical clearance from the Institutional Ethics Committee for proceeding with its research. The research obtained written consent from every participant before study enrollment. Data analysis occurred with SPSS software version 25.0. Descriptive statistics included mean and standard deviation statistics alongside frequency and percentage breakdowns to process the obtained data. The research examined group differences using the Chi-square and independent t-test at a p < 0.05 significance level.

## Results:

A total of 300 elderly participants were included in the study, with 150 from urban areas and 150 from rural areas. The mean age of the participants was 69.3 ± 4.5 years, with a male-to-female ratio of 1:1.2. Access to Dental Services Table 1 presents the distribution of participants based on their access to dental care. A significantly higher percentage of urban participants (62%) reported regular dental visits compared to only 26% in rural areas (p < 0.001). Additionally, dental insurance coverage was observed in 48% of urban participants but only 14% of rural participants. As shown in Table 1, rural participants reported transportation as a major barrier to accessing dental services (55%), compared to only 18% among their urban counterparts. The oral health status assessed using the DMFT index revealed a statistically significant difference between urban and rural participants (Table 2). The mean DMFT score was higher in rural areas (15.7 ± 3.8) than in urban areas (10.2 ± 3.1), indicating a greater burden of untreated dental disease in the rural population. As indicated in (Table 2), missing teeth were notably more prevalent among rural participants, contributing significantly to their higher DMFT scores (p < 0.01). Self-Perceived Oral Health and Barriers Table 3 summarize self-perceived oral health status and reported barriers. While 60% of urban participants rated their oral health as "good" or "very good," only 25% of rural participants did the same. Financial constraints and fear of dental procedures were common deterrents across both groups. As seen in Table 3, financial limitations were a more frequent concern in rural areas, potentially explaining the lower usage of dental services.

## Discussion:

This study establishes substantial differences between the oral health conditions and dental service provisions which exist between both urban and rural elderly demographic groups. The DMFT scores averaged higher among rural respondents while they lacked dental service availability and commonly reported financial constraints coupled with transportation difficulties. Research evidence shows that rural-residing older adults experience worse oral health results because of limited dental service availability combined with high prices [1, 2]. This research identified a crucial outcome that rural elderly people visited their dentist less often (26%) than urban seniors (62%). The shortage of dental facilities along with lengthy distances to services and limited transportation options determine why rural areas experience this discrepancy [4, 5]. Data from rural elderly adults (15.7 ± 3.8) accentuates the fact that treatment needs remain untreated in rural areas. Research has established that socioeconomic disadvantage affects the increased incidence of tooth loss because inadequate dental care services hinder access for elderly patients [6, 7]. The study revealed that urban participants showed higher fillings rates than their rural counterparts because they received better access to restorative dental services as previous research also indicated [8, 9]. Rural participants received dental insurance coverage at only 14% while urban participants had it at 48%. Consistent data shows dental insurance works as a significant factor that leads older people to visit the dentist regularly and receive treatment [10, 11. Multiple studies indicate that inadequate financial backing makes many elderly people choose not to receive dental care allowing dental diseases to become worse [12]. The rural population demonstrated an inferior assessment of their oral health condition. Rural residents evaluated their oral health condition worse than their urban counterparts as 25% rated it good while 60% in urban areas did so. Previous reports established that secure access to preventive dental care leads patients to rate their oral health condition better [13]. A fear of dental treatment and other psychological barriers existed equally in both populations suggesting older patients need particular attention when providing educational support and reassurance [14]. A comprehensive solution for these discrepancies requires primary care integration with oral healthcare and mobile dental services and senior citizen health insurance together with age-appropriate oral health education. The rural dental workforce needs expansion while service providers should receive incentives to work in underserved regions to reduce patient access limitations [15].

## Conclusion:

We need immediate policies authorizing specific dental care programs which address unique needs among elderly people dwelling in underprivileged areas.

## Figures and Tables

**Table 1:** Comparison of access to dental care between urban and rural participants (n = 300)

**Variable**	**Urban (n = 150)**	**Rural (n = 150)**	**p-value**
Regular dental visits (%)	62%	26%	<0.001
Dental insurance coverage (%)	48%	14%	<0.001
Transportation barrier (%)	18%	55%	<0.001

**Table 2:** DMFT scores among urban and rural elderly participants

**Location**	**Mean Decayed Teeth**	**Mean Missing Teeth**	**Mean Filled Teeth**	**Total DMFT Score (Mean ± SD)**
Urban (n=150)	2.1	5.3	2.8	10.2 ± 3.1
Rural (n=150)	3.8	9.6	2.3	15.7 ± 3.8
p-value	<0.05	<0.01	>0.05	<0.001

**Table 3:** Self-Perception of oral health and reported barriers (n = 300)

**Variable**	**Urban (%)**	**Rural (%)**	**p-value**
Self-rated oral health as good/very good	60%	25%	<0.001
Financial barrier to care	40%	66%	<0.001
Fear of dental treatment	32%	38%	>0.05
